# Phloridzin Docosahexaenoate, an Omega-3 Fatty Acid Ester of a Flavonoid Precursor, Inhibits Angiogenesis by Suppressing Endothelial Cell Proliferation, Migration, and Differentiation

**DOI:** 10.3390/biom14070769

**Published:** 2024-06-27

**Authors:** Wasundara Fernando, Emma MacLean, Susan Monro, Melanie R. Power Coombs, Paola Marcato, H. P. Vasantha Rupasinghe, David W. Hoskin

**Affiliations:** 1 Department of Pathology, Faculty of Medicine, Dalhousie University, Halifax, NS B3H 4R2, Canada; wasufer@dal.ca (W.F.); paola.marcato@dal.ca (P.M.); vrupasinghe@dal.ca (H.P.V.R.); 2 Department of Medical Sciences, Faculty of Medicine, Dalhousie University, Halifax, NS B3H 4R2, Canada; emma.maclean@dal.ca; 3 Department of Biology, Faculty of Science, Acadia University, Wolfville, NS B4P 2R6, Canada; susan.monro@acadiau.ca (S.M.); melanie.coombs@acadiau.ca (M.R.P.C.); 4 Department of Plant, Food, and Environmental Sciences, Faculty of Agriculture, Dalhousie University, Truro, NS B3H 4R2, Canada

**Keywords:** angiogenesis, flavonoid, fatty acid, esterification, signal transduction

## Abstract

Angiogenesis is a normal physiological process that also contributes to diabetic retinopathy-related complications and facilitates tumor metastasis by promoting the hematogenic dissemination of malignant cells from solid tumors. Here, we investigated the in vitro, ex vivo, and in vivo anti-angiogenic activity of phloridzin docosahexaenoate (PZ-DHA), a novel ω-3 fatty acid ester of a flavonoid precursor. Human umbilical vein endothelial cells (HUVEC) and human dermal microvascular endothelial cells (HMVEC) treated with a sub-cytotoxic concentration of PZ-DHA to assess in vitro anti-angiogenic activity showed impaired tubule formation on a Matrigel matrix. Ex vivo angiogenesis was measured using rat thoracic aortas, which exhibited reduced vessel sprouting and tubule formation in the presence of PZ-DHA. Female BALB/c mice bearing VEGF_165_- and basic fibroblast growth factor-containing Matrigel plugs showed a significant reduction in blood vessel development following PZ-DHA treatment. PZ-DHA inhibited HUVEC and HMVEC proliferation, as well as the migration of HUVECs in gap closure and trans-well cell migration assays. PZ-DHA inhibited upstream and downstream components of the Akt pathway and vascular endothelial growth factor (VEGF_165_)-induced overexpression of small molecular Rho GTPases in HUVECs, suggesting a decrease in actin cytoskeletal-mediated stress fiber formation and migration. Taken together, these findings reveal the potential of combined food biomolecules in PZ-DHA to inhibit angiogenesis.

## 1. Introduction

Angiogenesis, which is the formation of new blood vessels and capillaries from existing vasculature, depends on a complex and well-maintained balance between pro-angiogenic and anti-angiogenic factors. Angiogenesis occurs during wound healing, embryogenesis, and cyclic changes in the female reproductive system [1,2,3]. However, angiogenesis is also involved in disease states such as diabetic retinopathy, and the progression and metastasis of solid tumors [4,5].

Diabetic retinopathy is a secondary microvascular complication of diabetes mellitus caused by abnormal growth of retinal blood vessels, which is associated with multiple intraocular complications such as macular edema and choroidal neovascularization [6]. Cancer is another major disease that depends largely on angiogenesis for its progression. The supply of oxygen and nutrients is vital for the continuous growth and metastatic spread of solid tumors. In fact, without supporting vasculature, the size of a solid tumor is restricted to 1–2 mm^3^ [7]. The interior of a solid tumor is hypoxic as a result of the tumor vasculature being located, for the most part, around the tumor. This leads to the stabilization of the transcription factor, hypoxia-inducible factor-1 alpha (HIF-1α), leading to the activation of target genes such as vascular endothelial growth factor (VEGF) [8,9]. Although tumor hypoxia does not contribute to the unusual elevation of interstitial pressure in the tumor microenvironment [10], hypoxia-induced VEGF activation induces the growth of new blood vessels in the tumor interior [8,9]. Although the exact mechanisms and pathways that regulate the increased interstitial pressure in the tumor interior are not clearly delineated, current knowledge suggests that VEGF-induced formation of large numbers of leaky blood vessels with an irregular shape, as well as fibroblast-mediated tumor contractility, play a significant role [11,12]. In addition, many studies suggest that increased interstitial pressure within solid tumors restricts the delivery of chemotherapeutic drugs [11,13]. The formation of new blood vessels also serves as the main mechanism by which tumor cells enter the systemic circulation and travel to other sites in the body. Therefore, inhibition of angiogenesis is predicted to suppress cancer progression by limiting the metastatic spread of cancer cells [7,14].

Blocking receptors such as VEGFR2 prevents the binding of principal pro-angiogenic signaling molecules and is a common approach to inhibit angiogenesis [15]. Endothelial cell survival pathways such as Akt signaling have also been researched as therapeutic targets in the development of anti-angiogenic drugs [16]. Flavonoids demonstrate anti-angiogenic activity through the inhibition of Akt signaling and VEGFR2-mediated angiogenic signaling in endothelial cells [17,18]. Dietary ω-3 fatty acids such as docosahexaenoic acid (DHA) and its metabolites have proven to have anti-angiogenic activity [19,20]. Moreover, flavonoid-fatty acid conjugates show at least comparable, if not improved, anti-angiogenic activity when compared to parent flavonoids [21].

PZ-DHA combines a flavonoid precursor found in apple peels, known as phloridzin (PZ), with DHA through an enzyme-catalyzed acylation reaction. Supplementary Figure S1 shows the chemical structure of PZ-DHA. In our previous studies, we have shown that PZ-DHA possesses selective cytotoxic activity toward breast cancer cells, while sparing normal epithelial cells [22], and inhibits breast cancer cell metastasis in mice [23]. PZ-DHA also inhibits the growth of liver cancer cells [24] and T-cell acute lymphoblastic leukemia cells [25]. In this study, to investigate a potential mechanism for PZ-DHA-induced anti-metastatic activity, the impact of PZ-DHA on in vitro proliferation, migration, and tubule formation by human umbilical vein endothelial cells (HUVECs) and human microvascular vein endothelial cells (HMVECs) was tested. In addition, ex vivo anti-angiogenic activity was determined using thoracic aortic sections harvested from male Wistar rats. Furthermore, BALB/c female mice implanted with VEGF- and basic fibroblast growth factor (bFGF)-containing Matrigel plugs were used to evaluate the in vivo anti-angiogenic activity of systemically administered PZ-DHA. Finally, western blot analysis was employed to determine the effect of PZ-DHA on small molecular Rho GTPase signaling and phosphoinositide-dependent protein kinase 1 (PDK1), cyclin D3, and mammalian target of rapamycin (mTOR) activation.

## 2. Materials and Methods

### 2.1. Reagents and Chemicals

The horse radish peroxidase (HRP)/3,3′-Diaminobenzidine (DAB) detection system was purchased from Agilent Technologies (Mississauga, ON, Canada). Phenol red-free Matrigel was obtained from Corning Life Sciences (Tewkbury, MA, USA). The ECMatrix™ assay kit (EMD Millipore, Temecula, CA, USA) and endothelial basal medium (EBM)/supplements were purchased from Lonza Inc. (Walkersville, MD, USA). DHA was purchased from Nu-Chek Prep Inc. (Elysian, MN, USA). Human bFGF and human VEGF-165 were purchased from PeproTech (Rocky Hill, NJ, USA). RNase was obtained from Qiagen Inc. (Mississauga, ON, Canada). The Diff-Quik staining kit was purchased from Siemens Healthcare Diagnostics (Los Angeles, CA, USA). Aprotinin, 30% Brij 23 solution, Dulbecco’s Modified Eagle Medium (DMEM), Drabkin’s reagent, human hemoglobin, leupeptin, mitomycin C from *Streptomyces caespitosus*, Nonidet P-40 (NP-40), pepstatin A, phenazine methosulfate (PMS), phenylmethylsulfonyl fluoride (PMSF), PZ, porcine gelatin, sodium deoxycholate, and sodium fluoride (NaF) were purchased from Sigma-Aldrich (Oakville, ON, Canada).

### 2.2. Antibodies

Anti-CDK4 rabbit monoclonal Ab, anti-cyclinD3 rabbit monoclonal Ab, anti-total- PDK1 rabbit monoclonal antibody (Ab), anti-phospho-PDK1 (Ser241) rabbit monoclonal Ab, anti-total-mTOR rabbit monoclonal Ab, anti-phospho-mTOR (Ser2448) rabbit monoclonal Ab, anti-RhoA rabbit monoclonal Ab, anti-Rac1/2/3 rabbit monoclonal Ab, anti-Cdc42 rabbit monoclonal Ab, HRP-conjugated rabbit anti-β-actin, and HRP-conjugated donkey anti-rabbit Ab were purchased from Cell Signaling Technology Inc. (Danvers, MA, USA).

### 2.3. Cells and Cell Culture Conditions

HUVECs and HMVECs purchased from Lonza Inc. (Walkersville, MD, USA) were maintained in EBM, supplemented with fetal bovine serum, recombinant human bFGF, ascorbic acid, recombinant long R^3^-insulin-like growth factor 1, recombinant human epidermal growth factor, recombinant human VEGF, heparin, hydrocortisone, gentamicin sulfate, and amphotericin B, according to the supplier’s instructions. The cells were maintained at 37 °C in a humidified incubator containing 5% carbon dioxide. Cells were sub-cultured every 4–5 days.

### 2.4. Animals

Ethics approval for animal use was obtained from the Dalhousie University Committee on Laboratory Animals in accordance with Canadian Council for Animal Care guidelines. Six-to-eight-week-old female BALB/c female mice were purchased from Charles River Canada (Lasalle, QC, Canada). Mice were fed a regular rodent diet and water was supplied ad libitum.

### 2.5. Oregon Green 488 Staining

HUVECs and HMVECs were seeded into culture and synchronized. Adherent cells were stained with 1.25 µM Oregon Green 488 in serum-free DMEM for 45 min. The incubation was continued in a complete growth medium for 2 h to promote cell recovery. Cells were treated with sub-cytotoxic concentrations (10 or 20 µM) of PZ, DHA, PZ-DHA, vehicle, or medium alone and cultured in the dark for 72 h. Oregon Green 488 fluorescence of treated cells was measured using a FACSCalibur instrument (BD Biosciences, Mississauga, ON, Canada) in comparison to vehicle control and a non-proliferating control. The number of cell divisions (n) that took place was calculated using the formula: MCF_control_ = 2^n^ × MCF_treatment_ where MCF is the mean channel fluorescence.

### 2.6. Cell Cycle Analysis

Synchronized cells were seeded, and adherent cells were treated with a sub-cytotoxic concentration (10 µM) of PZ, DHA, PZ-DHA, vehicle, or medium alone, and cultured for 72 h. Cells were harvested, rinsed, and resuspended in ice-cold PBS. While vortexing, ice-cold 70% ethanol was added drop-by-drop and tube contents were incubated for at least 24 h at −20 °C to allow fixing. Fixed cells were washed with PBS and resuspended in cell cycle staining solution (0.1% *v*/*v* Triton-X-100 and 2 μL/mL DNA-free Rnase A in 1 × PBS) containing 20 μL/mL propidium iodide and incubated for 30 min at room temperature. Flow cytometric analysis was performed using a FACSCalibur instrument.

### 2.7. Gap Closure Assays

HUVECs and HMVECs (10,000 cells/100 µL) were seeded in 2-well culture inserts (Ibidi GmbH, Martinsried, Germany) placed in 6-well plates and adherent cells were treated with 10 µg/mL mitomycin C in serum-free DMEM medium for 2 h at 37 °C to inhibit cell proliferation. Cells were allowed to recover for 12 h in EBM-containing serum and treated with PZ, DHA, PZ-DHA (10 µM), vehicle, or medium alone for 24 h. Culture inserts were removed, and the gaps were periodically photographed, starting at t = 0 h, until completely closed by medium-treated cells (t = 20 h).

### 2.8. Trans-Well Cell Migration Assay

HUVECs were seeded and treated with 10 μM of PZ, DHA, PZ-DHA, vehicle, or medium alone for 24 h, and treatments were continued in serum-free medium for another 6 h. Cells (50,000) were resuspended in 50 µL warm serum-free medium and loaded into the wells of the top chamber of co-culture inserts (Thermo Fisher Scientific, Mississauga, ON, Canada). Cells were allowed to migrate through an 8 µm porous membrane for 22 h. Migrated cells were stained using a Diff-Quik™ staining set.

### 2.9. Western Blot Analysis

Adherent HUVECs were treated with 10 μM of PZ, DHA, PZ-DHA, vehicle, or medium alone for 72 h. Cells were harvested and incubated in ice-cold lysis buffer [50 mM Tris (pH 7.5), 150 mM sodium chloride, 50 mM disodium hydrogen phosphate, 0.25% sodium deoxycholate (*w*/*v*), 0.1% NP-40 (*v*/*v*), 100 μM sodium orthovanadate, 10 mM NaF, 5 mM ethylenediaminetetraacetic acid, and 5 mM ethylene glycol tetraacetic acid containing freshly added protease inhibitors (1 mM PMSF, 10 μg/mL aprotinin, 5 μg/mL leupeptin, 10 μM phenylarsine oxide, 1 mM dithiothreitol, and 5 μg/mL pepstatin) for 15 min. Cell lysates were clarified by centrifugation and the protein concentration was determined by Bradford assay. After electrophoresis, proteins were transferred to nitrocellulose membranes and blots were incubated in 5% non-fat milk or 5% BSA for 1 h at room temperature to block nonspecific binding. Blots were probed overnight at 4 °C with primary Ab against the protein of interest. Then, the blots were washed thoroughly with Tween-TBS and probed with HRP-conjugated donkey anti-rabbit or goat anti-mouse IgG Ab for 1 h at RT. Even protein loading was confirmed by probing the blots with HRP-conjugated rabbit anti-β actin Ab or HRP-conjugated rabbit anti-α tubulin Ab. The proteins of interest were visualized by a ChemidocTouch™ imaging system (Bio-Rad Laboratories, Mississauga, ON, Canada).

### 2.10. In Vitro Angiogenesis Assay

In vitro angiogenesis was studied using a commercially available in vitro angiogenesis ECMatrix™ assay kit (EMD Millipore, Temecula, CA, USA), according to the manufacturer’s instructions. Briefly, 9 parts of ECMatrix were mixed with one part of the diluent buffer and 10 µL of the mixture was added to the inner well of the µ-slide angiogenesis plate (Ibidi GmbH, Martinsried, Germany). The plate was incubated at 37 °C for 1 h. HUVECs or HMVECs (7500 cells) treated with 10 µM (HMVECs) or 20 µM (HUVECs) PZ, DHA, PZ-DHA, vehicle, or medium alone for 72 h were resuspended in 50 µL of EGM and seeded onto polymerized ECMatrix. Tubule formation by HMVECs and HUVECs was monitored and photographed after 4 h and 6 h, respectively. Supplementary Table S1 shows the scoring scheme.

### 2.11. Ex Vivo Angiogenesis Assay

Aortas from adult male Wistar rats were cleaned using sterile saline and cut into 1 mm × 3 mm sections. Aorta sections were then embedded in 200 μL Matrigel and incubated at 37 °C for 1 h. Matrigel was covered with 200 µL EBM and incubated overnight at 37 °C (day 0). On Day 1, aorta sections embedded in Matrigel were treated with PZ, DHA, PZ-DHA (20 µM), vehicle, or medium alone for 8 days. Medium/treatment was changed on Day 4. The development of tubules from aortic endothelium was monitored and photographed on Days 5 and 8.

### 2.12. Matrigel Plug Assay

Phenol red-free Matrigel (300 µL) containing human VEGF_165_ (2 µg/mL) and bFGF (2 µg/mL) was implanted by subcutaneous injection on both the left and right sides along the mid-dorsal line of the lower posterior area of BALB/c female mice (Day 0). On Day 1, mice were randomly assigned into two groups (11 mice/group) and 5 doses of saline or PZ-DHA (100 mg/kg) were administered by intraperitoneal injection every second day for 9 days. After 9 days, Matrigel plugs were harvested and photographed. Hemoglobin concentration in Matrigel plugs was determined using the cyanmethemoglobin method. Briefly, a human hemoglobin (0.717 mg/mL) solution made in Drabkin’s reagent (Sigma-Aldrich, Oakville, ON, Canada) containing 0.0005% *v*/*v* 30% Brij 23 solution was used to generate the standard curve of cyanmethemoglobin (R^2^ = 1.00). Matrigel plugs were homogenized in 500 µL of Drabkin’s reagent and homogenates were centrifuged at 9600× *g* at 4 °C for 6 min. The supernatant was collected and transferred into 96-well plates in triplicate. Absorbance was measured at 540 nm and cyanmethemoglobin concentration in Matrigel plugs was calculated.

### 2.13. Statistical Analysis

Three independent experiments were performed for each assay and the mean of the three experiments was calculated. A one-way ANOVA multiple means comparison method was performed and the differences between means were compared using Tukey’s post-mean comparison method. The analysis was considered significant at the following levels: * *p* < 0.05, ** *p* < 0.01, *** *p* < 0.001.

## 3. Results

### 3.1. PZ-DHA Inhibits Angiogenesis In Vitro and Ex Vivo

**Figure 1:**
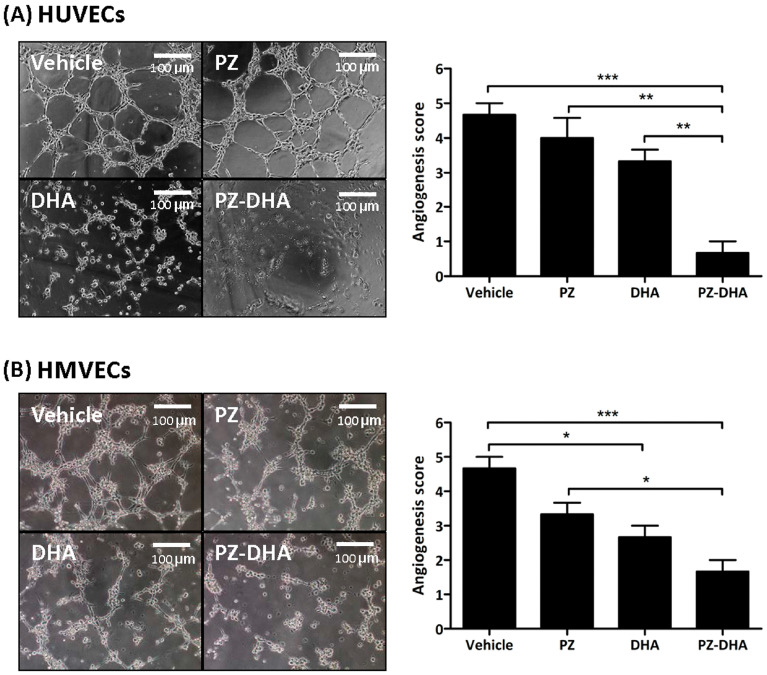
PZ-DHA inhibits angiogenesis in vitro. PZ, DHA, PZ-DHA (HUVECs: 20 µM, HMVECs: 10 µM), vehicle-, or medium-treated HMVECs or HUVECs were harvested, and 7500 cells were resuspended in 50 µL of EGM and seeded onto a polymerized ECMatrix. (**A**) HMVEC and (**B**) HUVEC tubule formation were monitored and photographed after 4 h and 6 h, respectively. Images were analyzed and tube formation was quantified according to the complexity of the tube network. Mean angiogenesis scores ± SEM were calculated from 3 independent experiments. Statistical analysis was performed using the ANOVA multiple means comparison method and differences among means were compared using Tukey’s post-mean comparison method; * *p* < 0.05, ** *p* < 0.01, and *** *p* < 0.001.

The concentrations of PZ, DHA, and PZ-DHA for HUVECs and HMVECs used in this study were confirmed to be sub-cytotoxic using 7AAD cell viability assays (Supplementary Figure S2). This was to ensure that there would be no inadvertent activation of cell death pathways while determining the impact of PZ-DHA on angiogenesis. In vitro angiogenesis was scored according to the stage/complexity of the tubule formation by HUVECs and HMVECs treated with vehicle, PZ, DHA, or PZ-DHA. Treatment with PZ-DHA (20 μM) decreased the in vitro HUVEC angiogenesis by 6.5-fold; however, PZ alone did not inhibit tubule formation by HUVECs (Figure 1A). The inhibitory effect of PZ-DHA was significantly greater than either PZ or DHA alone. PZ-DHA (10 μM), as well as DHA, significantly attenuated the in vitro tubule formation by HMVECs by 2.8-fold and 1.8-fold, respectively (Figure 1B). Again, PZ-DHA had a greater impact on tubule formation than PZ or DHA alone. In preliminary experiments, PZ-DHA also suppressed ex vivo angiogenesis. As shown in Supplementary Figure S3, the sprouting of microvessels from rat aorta endothelium embedded in a Matrigel matrix was reduced in the presence of 20 μM PZ-DHA. Upon extended incubation, PZ-DHA-treated cells aligned on the Matrigel matrix but did not differentiate to form tubules.

### 3.2. PZ-DHA Inhibits In Vivo Angiogenesis in BALB/c Female Mice

**Figure 2:**
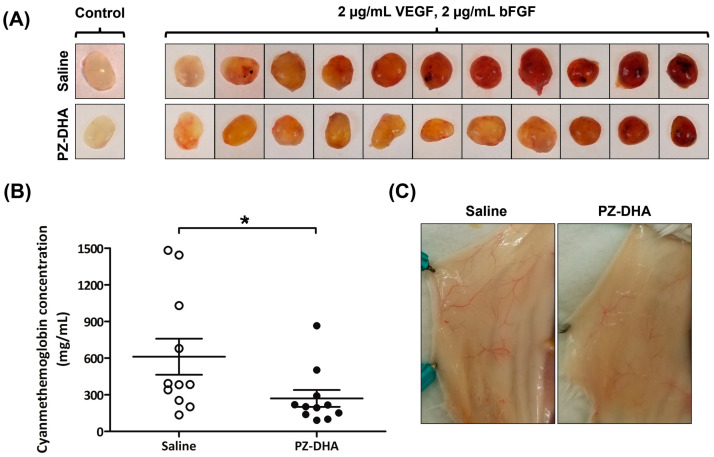
PZ-DHA inhibits in vivo angiogenesis in female BALB/c mice. Phenol red-free Matrigel (300 µL) mixed with/without human VEGF_165_ (2 µg/mL) and bFGF (2 µg/mL) were subcutaneously implanted on both the left and right sides along the mid-dorsal line of the lower posterior area of BALB/c female mice (Day 0). On Day 1, mice were randomly assigned into 2 groups (11 mice/group) and saline or PZ-DHA (100 mg/kg) was administered by intraperitoneal injection. Altogether, saline or PZ-DHA was administered every second day (Days 1, 3, 5, 7, and 9) for 9 days. Mice were euthanized. (**A**) Matrigel plugs were harvested and photographed. Control Matrigel plugs are shown to confirm that angiogenesis did not occur in the absence of VEGF_165_ and bFGF. (**B**) Mean hemoglobin concentration ± SEM of VEGF_165_- and bFGF-containing Matrigel plugs from saline- and PZ-DHA-treated mice was determined using the cyanmethemoglobin method. Statistical differences between means were compared using Student’s *t*-test; * *p* < 0.05. (**C**) Growth of blood vessels on the body wall, toward the Matrigel plugs, was also photographed.

The impact of PZ-DHA on in vivo angiogenesis was investigated using a Matrigel plug assay performed in BALB/c female mice. VEGF- and bFGF-induced angiogenesis in Matrigel plugs was inhibited by intraperitoneal administration of PZ-DHA (Figure 2A). Hemoglobin in Matrigel plugs was converted into a cyanmethemoglobin complex by a reaction with cyanide ions in Drabkin’s reagent. A 2.3-fold reduction in the formation of cyanmethemoglobin complex was noted in Matrigel plugs excised from PZ-DHA-treated mice (Figure 2B). PZ-DHA-induced reduction in the growth of blood vessels in the body wall (around the Matrigel plugs) was also observed (Figure 2C).

### 3.3. PZ-DHA Inhibits the Proliferation of HUVECs and HMVECs

**Figure 3:**
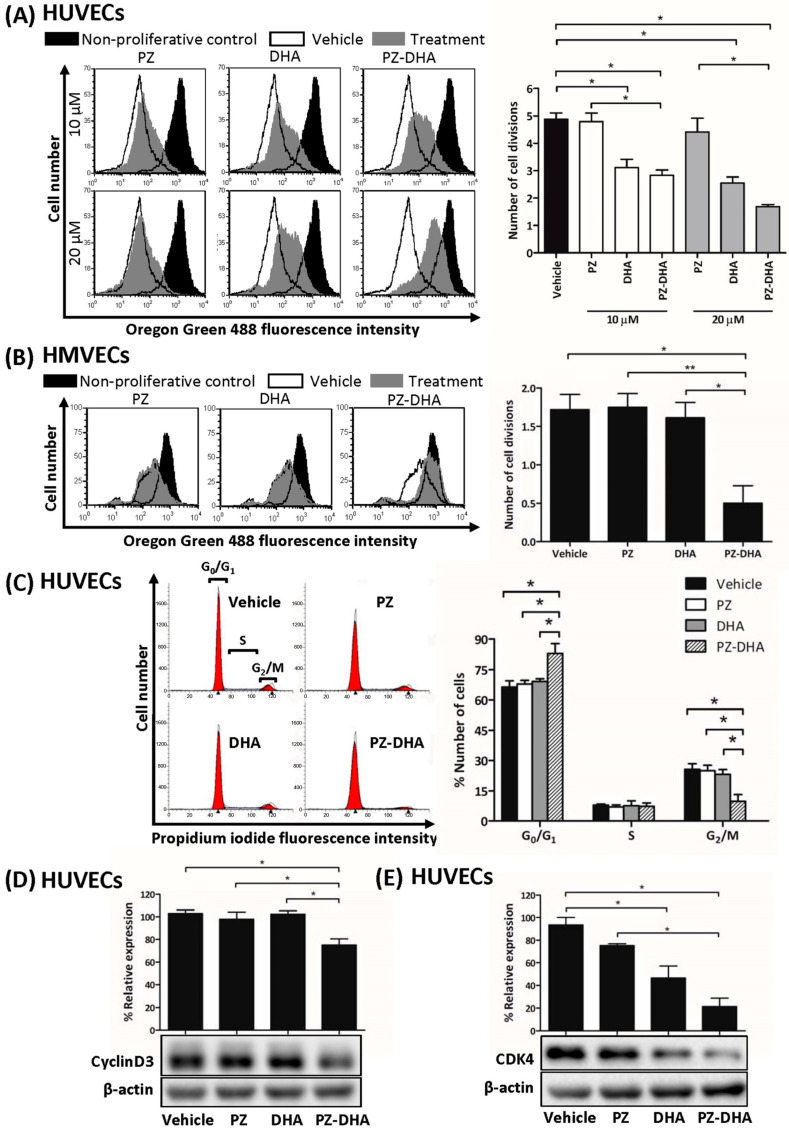
PZ-DHA inhibits the in vitro proliferation of HUVECs and HMVECs. Oregon Green 488-stained (**A**) HUVECs and (**B**) HMVECs were treated with PZ, DHA, PZ-DHA (HUVECs: 10 or 20 µM, HMVECs: 10 µM), vehicle, or medium and cultured for 72 h at 37 °C. At the end of incubation, cells were harvested and analyzed by flow cytometry. Data shown are representative histograms and mean number of cell divisions ± SEM. (**C**) HUVECs were treated with PZ, DHA, PZ-DHA (10 µM), vehicle, or medium alone and cultured for 72 h at 37 °C. Cells were fixed and stained with PI in the presence of RNase for analysis by flow cytometry. Representative histograms were generated and the mean % number ± SEM of cells in each phase of the cell cycle was calculated. (**D**) Cyclin D3 and (**E**) CDK4 expression were determined using western blot analysis of protein-rich cell lysates HUVECs that were treated with PZ, DHA, PZ-DHA (10 µM), vehicle, or medium alone for 72 h. Statistical analysis of data from 3 independent experiments was performed using the one-way ANOVA multiple means comparison method, and differences among means were compared using Tukey’s post-mean comparison method. * *p* < 0.05, ** *p* < 0.01. (Original Western Blot Images see supplementary Figure S4).

We next determined the mechanism(s) that might account for the inhibitory effect of PZ-DHA on in vitro, ex vivo, and in vivo angiogenesis. As shown in Figure 3A, 10 μM PZ-DHA decreased HUVEC proliferation by 1.7-fold, and by 2.8-fold when HUVECS were treated with 20 μM PZ-DHA. DHA alone also had an antiproliferative effect on HUVECs. PZ-DHA at 10 μM suppressed HMVEC proliferation by 3.4-fold (Figure 3B). Neither DHA nor PZ alone affected HMVEC proliferation. Figure 3C shows that 10 μM PZ-DHA arrested the HUVEC cell cycle at G_0_/G_1_ while significantly decreasing the number of cells progressing to the G_2_/M phase. The progression of a cell through G1 phase of the cell cycle is regulated by several cyclins and CDKs, including cyclin D and CDK4. PZ-DHA (10 μM) significantly decreased cyclin D3 levels in HUVECs by 28% (Figure 3D), and the expression of CDK4 (Figure 3E) was suppressed by both DHA and PZ-DHA by 47% and 72%, respectively; however, PZ by itself had no effect on HUVEC and HMVEC proliferation or expression of any of the cell cycle regulatory proteins tested. These findings suggest that the antiproliferative effect of PZ-DHA on endothelial cells contributes to its anti-angiogenic activity.

### 3.4. PZ-DHA Inhibits the Migration of HUVECs and HMVECs

**Figure 4:**
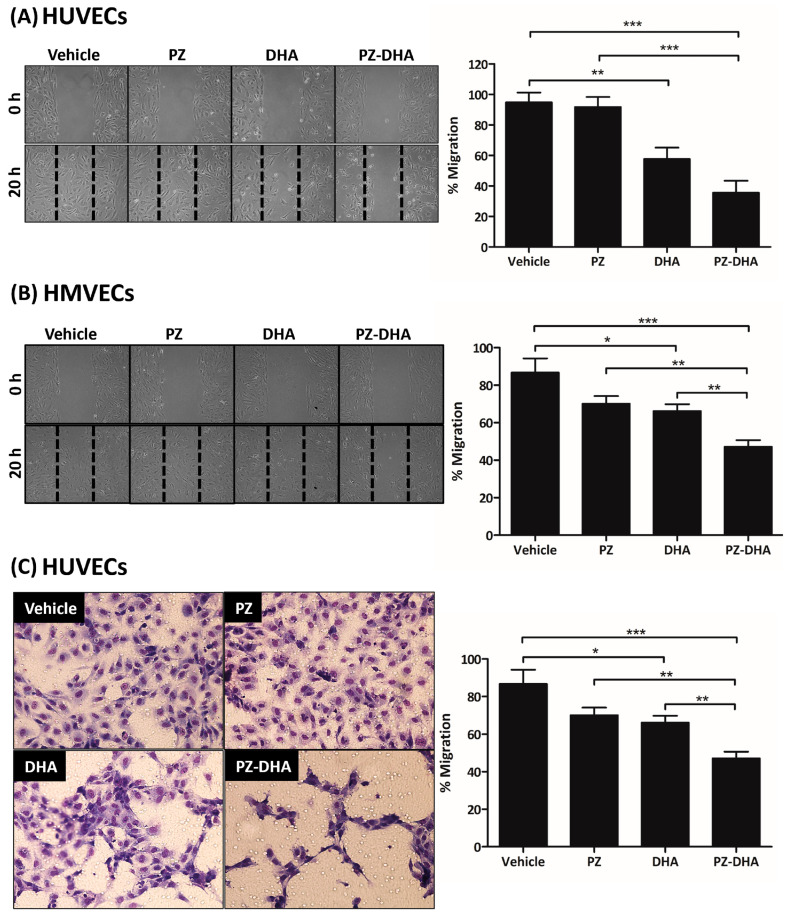
PZ-DHA inhibits the in vitro migration of HUVECs and HMVECs. (**A**) HMVECs and (**B**) HUVECs in cell culture inserts were treated with PZ, DHA, PZ-DHA (10 μM), vehicle, or medium alone and cultured for 24 h. Inserts were removed and the number of cells that migrated into the gap was quantified. Representative pictures of cells in the gap and mean % migrated cells ± SEM are shown. (**C**) HUVECs were seeded and treated with PZ, DHA, PZ-DHA (10 μM), vehicle, or medium alone and cultured for 24 h. Treated cells were serum-starved and migration toward serum through a porous membrane was determined. Representative pictures of migrated cells and mean % migration ± SEM are shown. Statistical analysis of data from 3 independent experiments was carried out using the ANOVA multiple means comparison statistical method and differences among means were compared using Tukey’s test; * *p* < 0.05, ** *p* < 0.01, *** *p* < 0.001.

Endothelial cell migration is important during early angiogenesis and subsequent tubule formation [26,27]. PZ-DHA (10 µM) reduced the migration of HUVECs and HMVECs by 59% (Figure 4A) and 56% (Figure 4B), respectively, in a gap closure assay. PZ-DHA-induced inhibition of HUVEC migration was further investigated using a trans-well cell migration assay. The migration of serum-starved-HUVECs showed a significant 40% inhibition in the presence of 10 µM PZ-DHA. This inhibitory effect of PZ-DHA was significantly greater than that of either parent compound; however, the polygonal endothelial cellular shape was restored by vehicle-, PZ-, and DHA-treated HUVEC following migration. In contrast, the shape of PZ-DHA-treated HUVECs was still distorted after migration (Figure 4C). These data suggest the inhibitory effect of PZ-DHA on endothelial cell migration is a factor in its anti-angiogenic activity.

### 3.5. PZ-DHA Inhibits PDK1 and mTOR Activation and VEGF_165_-Induced Small Molecular Rho GTPase Signaling in HUVECs

**Figure 5:**
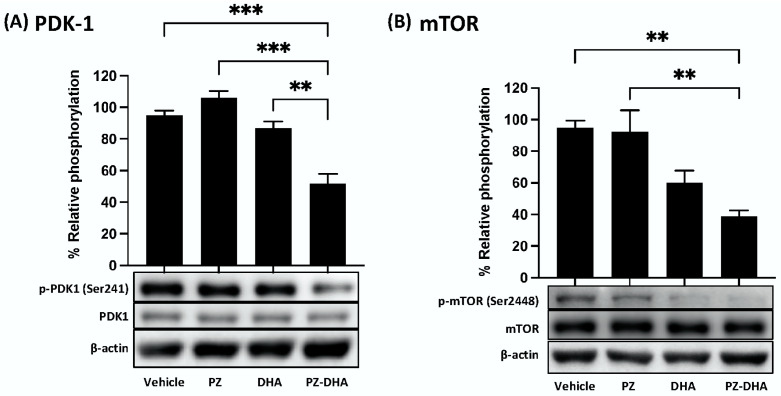
PZ-DHA inhibits PDK1 and mTOR phosphorylation in HUVECs. HUVECs were treated with PZ, DHA, PZ-DHA (10 μM), or vehicle alone and cultured for 72 h. Cells were harvested and protein-rich cell lysates were prepared for western blot analysis of (**A**) phospho-PDK1 (Ser241)/total-PDK1 expression or (**B**) phospho-mTOR (Ser2448)/total-mTOR expression. Equal protein loading was confirmed by β-actin expression. Data shown are mean % relative expression ± SEM from 3 independent experiments. The ANOVA multiple means comparison statistical method was performed and differences among means were compared using Tukey’s test; ** *p* < 0.01, *** *p* < 0.001. (Original Western Blot Images see supplementary Figure S5).

The PDK1-activated phosphatidylinositol 3-kinase (PI3K)/Akt/mTOR pathway is involved in blood vessel growth and maintenance, which also involves Rho GTPase signaling, which is often activated by VEGF_165_ [28,29,30]. PZ-DHA inhibited upstream (PDK1) and downstream (mTOR) components of the Akt signaling pathway in HUVECs. As shown in Figure 5A, 10 μM PZ-DHA inhibited the phosphorylation of PDK1 at Ser241. The downstream phosphorylation of mTOR at Ser2448 was also decreased in the presence of 10 μM PZ-DHA (Figure 5B).

**Figure 6:**
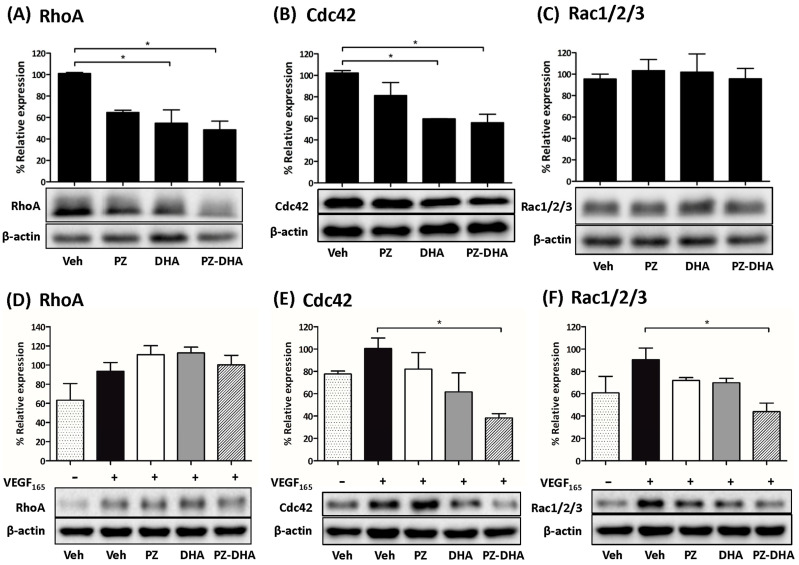
PZ-DHA inhibits endogenous and VEGF-induced small GTPase signaling in HUVECs. HUVECs were treated with PZ, DHA, PZ-DHA (10 μM), or vehicle alone and cultured for 72 h in the presence or absence of VEGF_165_. Cells were harvested and protein-rich cell lysates were prepared for western blot analysis of (**A**,**D**) RhoA expression, (**B**,**E**) Cdc42 expression, and (**C**,**F**) Rac1/2/3 expression. Equal protein loading was confirmed by β-actin expression. Data shown are mean % relative expression ± SEM from 3 independent experiments. The ANOVA multiple means comparison statistical method was performed and differences among means were compared using Tukey’s test; * *p* < 0.05. (Original Western Blot Images see supplementary Figure S6).

Figure 6A shows that 10 μM PZ-DHA, as well as the same concentration of DHA, significantly inhibited RhoA expression in HUVECs (Figure 6A). Cdc42 expression was also decreased following 10 μM PZ-DHA or DHA treatment (Figure 6B). In contrast, Rac1/2/3 expression remained unchanged in the presence of 10 μM PZ-DHA (Figure 6C). VEGF_165_-induced RhoA activation was not reduced in the presence of 10 μM PZ, DHA, or PZ-DHA (Figure 6D); however, VEGF_165_-induced Cdc42 overexpression was reduced by 10 μM PZ-DHA (Figure 6E). VEGF_165_-induced Rac1/2/3 expression was also significantly inhibited in the presence of 10 μM PZ-DHA, although there was no effect by either parent compound alone (Figure 6F). These findings suggest that PZ-DHA-mediated inhibition of key angiogenesis-associated signal transduction pathways is associated with its anti-angiogenic activity.

## 4. Discussion

Angiogenesis plays an important role in several pathological conditions including chronic inflammation and cancer [4,5]. In the current study, the effects of PZ-DHA on different aspects of angiogenesis, including endothelial cell proliferation, migration, and differentiation, were tested in vitro, ex vivo, and in vivo. PZ-DHA attenuated in vitro tubule formation by HUVECs and HMVECs, microvessel sprouting from rat aortic endothelium, and VEGF- and bFGF-induced angiogenesis in Matrigel plugs implanted in BALB/c mice. These findings are consistent with anti-angiogenic effects of other flavonoids such as quercetin [31], apigenin [32], and epigallocatechin gallate [33], which suppress angiogenesis via their inhibition of Akt phosphorylation, HIF-1α signaling, and expression of cell adhesion molecules.

The proliferation of HUVECs and HMVECs was attenuated by sub-cytotoxic concentrations of PZ-DHA, causing inhibition of S phase entry of HUVECs because of G_0_/G_1_ cell cycle arrest. At a molecular level, the expression of cyclin D3 and CDK4 was inhibited by PZ-DHA, suggesting a decreased formation of cyclin D/CDK4 complex. This observation was in line with the increased accumulation of HUVECs in the G_0_/G_1_ phase. DHA, one of the parent compounds, did not alter the expression of cyclin D3 or the number of HUVECs passing the G1 checkpoint to enter the S phase; however, the expression of CDK4 was downregulated by DHA. In contrast, Kim and colleagues showed that DHA induces G_0_/G_1_ cell cycle arrest, causes an increase in the sub-G_1_ peak, and increases staining with Annexin-V-FITC/PI, all of which are consistent with HUVEC apoptosis [34]. Our findings differ, possibly because an apoptosis-inducing concentration (40 μM) of DHA was used by Kim et al., whereas our study was conducted using sub-cytotoxic concentrations of DHA (10–20 μM). The fate of a cell during stress conditions is determined by an interplay of cell cycle regulators and pro-apoptotic factors [35,36]. Induction of apoptotic signals vis p53 activation may have played a partial role through the p53-p21 axis in the G_0_/G_1_ cell cycle arrest [34]. G_1_/S transition is also regulated by a type of GTPase family protein known as Rho [37], which was inhibited by PZ-DHA. Furthermore, the accumulation of p21^WAF1/CIP1^ inhibits CDK activity, which negatively regulates the G_1_/S transition [38]. In summary, both the inhibition of cell cycle regulatory proteins and small molecular Rho GTPase molecules have been attributed to PZ-DHA-induced anti-proliferative activity in endothelial cells.

Rho GTPase signaling regulates endothelial cell rearrangement and organization during angiogenesis [39,40]. VEGF_165_-dependent Rac activation via Rac1 guanine nucleotide exchange factor DOCK4 is necessary for Cdc42 activation, filopodia, and lumen formation [41]. PZ-DHA down-regulated VEGF_165_-induced Cdc42 and Rac1/2/3 expression by HUVECs but did not affect the expression of RhoA in response to VEGF_165_. However, PZ-DHA significantly decreased tubule formation by HUVECs and HMVECs in vitro. Preliminary experiments suggest that PZ-DHA also inhibited VEGF_165_-induced sprouting of tubules from rat aortic sections ex vivo. These findings indicate that PZ-DHA-induced anti-angiogenic activity was not necessarily mediated solely through RhoA-dependent mechanisms, but possibly in combination with inhibition of VEGF_165_-stimulated Cdc42 and Rac1/2/3.

PZ-DHA inhibited the migration and expression of RhoA and Cdc42, but not Rac1/2/3, in HUVECs, suggesting inhibition of filopodia-driven HUVEC migration. PZ-DHA significantly and more effectively suppressed the migration of HUVECs in trans-well cell migration assays, compared to its parent compounds. The polygonal cellular shape was restored following the migration of vehicle-, PZ-, and DHA-treated HUVEC; but not in PZ-DHA-treated cells. The process of angiogenesis is composed of multiple steps that include changes in the integrity of the endothelial cell barrier, basement membrane degradation, endothelial cell proliferation and migration, morphogenesis, and capillary formation, all of which involve distinct roles by RhoA, Cdc42, and Rac1/2/3 [42]. Well-known cell survival and proliferation pathways such as the PDK1-activated PI3K/Akt/mTOR pathway and mitogen-activated protein kinase signaling pathways regulate endothelial cell proliferation and assembly [43,44]. Crosstalk between cell survival pathways and other signaling cascades, such as Rho GTPase and matrix metalloproteinase signaling, regulate angiogenesis [45,46].

Induction of the PDK1-activated PI3K/Akt/mTOR pathway impacts multiple downstream signal transduction pathways involved in endothelial cell proliferation (cell cycle regulators), migration through actin reorganization (small molecular RhoGTPases), and eventual blood vessel development [47,48,49]. At a sub-cytotoxic concentration, PZ-DHA suppressed PDK1 phosphorylation upstream of Akt and cyclin D3 and mTOR phosphorylation downstream from Akt. Currently, it is not known whether the phosphorylation of endothelial cell Akt is directly inhibited by PZ-DHA. However, the DHA component of PZ-DHA inhibits the phosphorylation of Akt at ser473 and thr308, as well as mTOR phosphorylation, in human prostate cancer cells [50].

**Figure 7:**
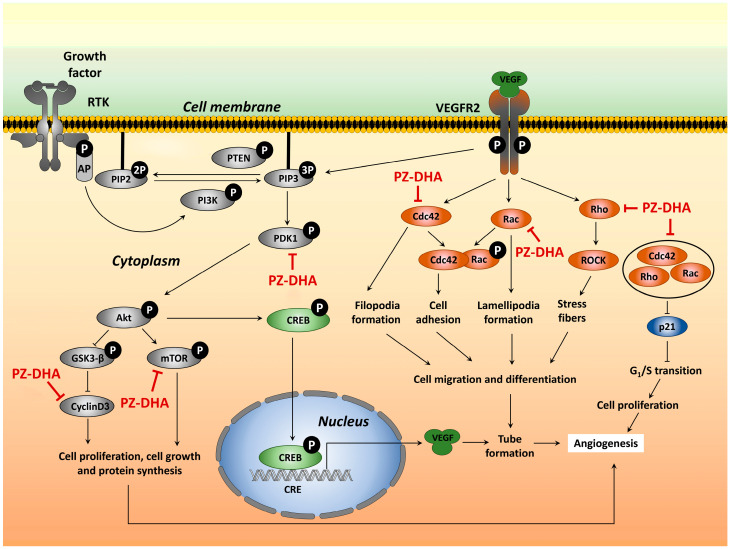
Proposed scheme for the anti-angiogenic activities of PZ-DHA. PZ-DHA inhibits angiogenesis by inhibiting endothelial cell proliferation, migration, and tubule formation. The antiproliferative effects of PZ-DHA on endothelial cells are mediated through Rho GTPase-driven G_1_ arrest through the p53-p21 axis, as well as inhibition of upstream and downstream components of the PI3K/Akt/mTOR signaling pathway. PZ-DHA inhibits endothelial cell migration and differentiation by inhibiting VEGF-induced activation of small molecular Rho GTPase and activation of PDK1, cyclin D3, and mTOR. Akt: protein kinase B; AP: activator protein; Cdc42, Rac1/2/3, and RhoA: Rho family small GTPases; CRE: cAMP (cyclic adenosine monophosphate) response element; CREB: cAMP response element binding protein; GSK3-β: glycogen synthase kinase 3-β; mTOR: mammalian target of rapamycin; p21: cyclin-dependent kinase inhibitor 1; PDK1: phosphoinositide-dependent kinase-1; PI3K: phosphoinositide 3-kinase; PIP2: phosphatidylinositol-4,5-bisphosphate; PIP3: phosphatidylinositol-3,4,5-trisphosphate; PTEN: phosphatase and tensin homolog; PZ-DHA: phloridzin docosahexaenoate; ROCK: Rho-associated protein kinase; RTK: receptor tyrosine kinase; VEGF: vascular endothelial growth factor; VEGFR2: vascular endothelial growth factor receptor.

Figure 7 depicts a model for the impact of PZ-DHA on angiogenesis-associated signal transduction that is consistent with our findings. The effect of PZ-DHA at the receptor binding level is yet to be understood.

Irregularity in shape, high permeability, and poorly supported pericytes are key features of tumor-associated blood vessels [51,52]. This leads to the formation of leaky and contorted vasculature, which is associated with the increased interstitial fluid pressure within the tumor that facilitates passive migration of cancer cells and poor penetration of the tumor by chemotherapeutic drugs [11,13]. Although it was initially thought that angiogenesis blockade eliminates cancer cells by oxygen and nutrient starvation, recent experience suggests that “normalization” of tumor-associated angiogenesis is more effective than its inhibition since this approach corrects elevated interstitial fluid pressure, thereby allowing chemotherapeutic drug penetration and reduced cancer cell metastasis [53]. PZ-DHA-mediated inhibition of upstream (PDK1) and downstream (mTOR) components of the Akt signaling pathway may correct the abnormal vasculature in tumors since inhibition of PI3K/Akt/mTOR signaling promotes endothelial cell elongation associated with proper regulation of angiogenesis [54]. However, confirmation awaits further investigation of the effect of PZ-DHA on abnormal angiogenesis in tumors. We conclude that in addition to targeting breast cancer cells [23], PZ-DHA may also interfere with the angiogenesis-related hematogenic spread of breast cancer cells, thereby contributing to reduced metastasis of PZ-DHA-treated mammary carcinoma cells in a mouse model of metastatic breast cancer [23].

## Data Availability

Data available on request.

## Supplementary Materials

The following supporting information can be downloaded at: https://www.mdpi.com/article/10.3390/biom14070769/s1, Figure S1: Chemical structure of PZ-DHA; Figure S2: Determination of subcytotoxic concentrations of PZ-DHA for HMVECs and HUVECs in vitro; Figure S3: PZ-DHA inhibits angiogenesis ex vivo; Table S1: Angiogenesis pattern and scoring. Figures S4–S6: Original western blot images for Figure 3, Figure 5 and Figure 6.
