# Supplementary material for: Phloridzin Docosahexaenoate, an Omega-3 Fatty Acid Ester of a Flavonoid Precursor, Inhibits Angiogenesis by Suppressing Endothelial Cell Proliferation, Migration, and Differentiation
Source: Biomolecules. 2024 Jun 27;14(7):769. doi: 10.3390/biom14070769 (PMC11274491; doi:10.3390/biom14070769)
Supplement: Supplementary file 1 [file biomolecules-14-00769-s001.zip › biomolecules-2653714-supplementary.pdf]

Supplementary Material

Table S1. Angiogenesis pattern and scoring

Pattern of endothelial cell arrangement	Angiogenesis score
Well separated individual cells	0
Cells begin to migrate and align themselves	1
Capillary tubes visible; no sprouting	2
Sprouting of new capillary tubes visible	3
Closed polygons begin to form	4
Complex mesh-like structures develop	5

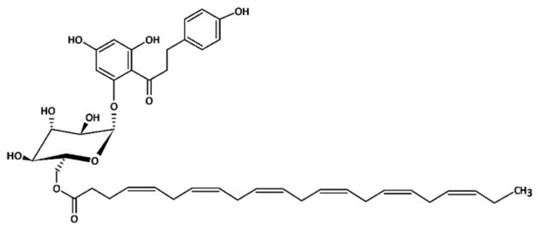

Figure S1. Chemical structure of PZ-DHA

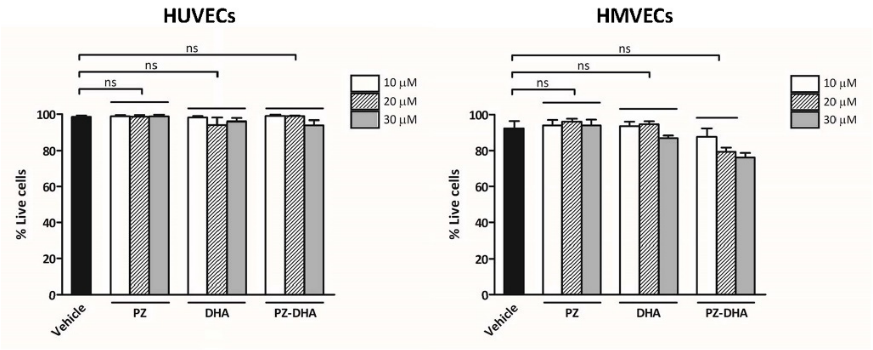

Figure S2. Determination of sub-cytotoxic concentrations of PZ-DHA for HMVECs and HUVECs in vitro.

HMVECs and HUVECs were seeded and treated with 10, 20, or 30 µM PZ, DHA, PZ-DHA, vehicle or medium alone and cultured for 72 h at 37°C Cells were then harvested and stained with 0.525 µg/mL 7-aminoactinomycin D for 5 min at room temperature and the live cell population was identified by reading samples at FL3 detector of the flow cytometer. Data shown are mean % live cells±SEM of 4 independent experiments; ns: not significant.

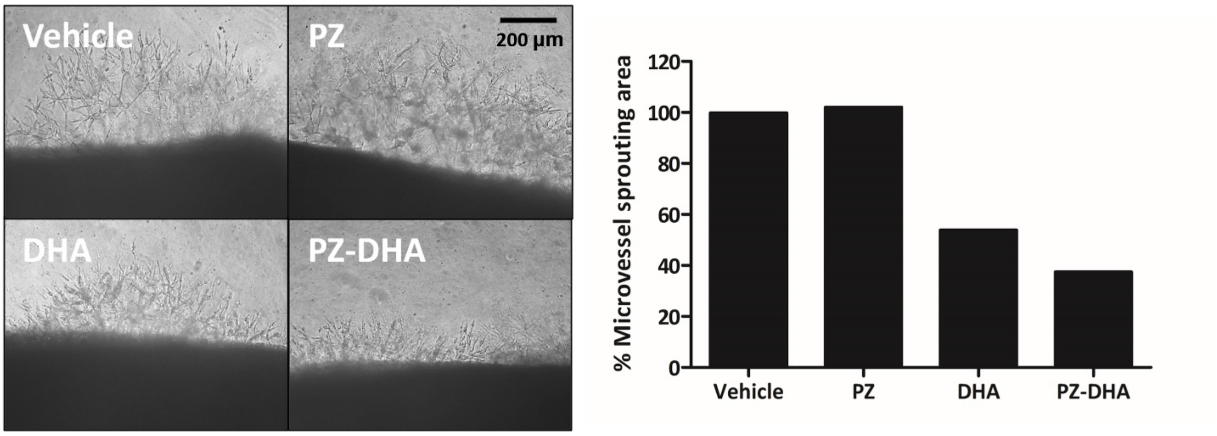

Figure S3. PZ-DHA inhibits angiogenesis ex vivo. Sectioned thoracic aortas from adult male Wistar rats were embedded in Matrigel and covered with 200 µL EGM-2 supplemented with VEGF and bFGF containing PZ, DHA, PZ-DHA (20 µM), vehicle, or medium alone and cultured for 8 days. Sprouting and branching of endothelial cells from the aortas are shown. Scale bar shown is the same for all images. The surface area covered by sprouted microvessels was calculated. Data shown are the mean microvessel sprouting area from 2 independent experiments that yielded comparable results.

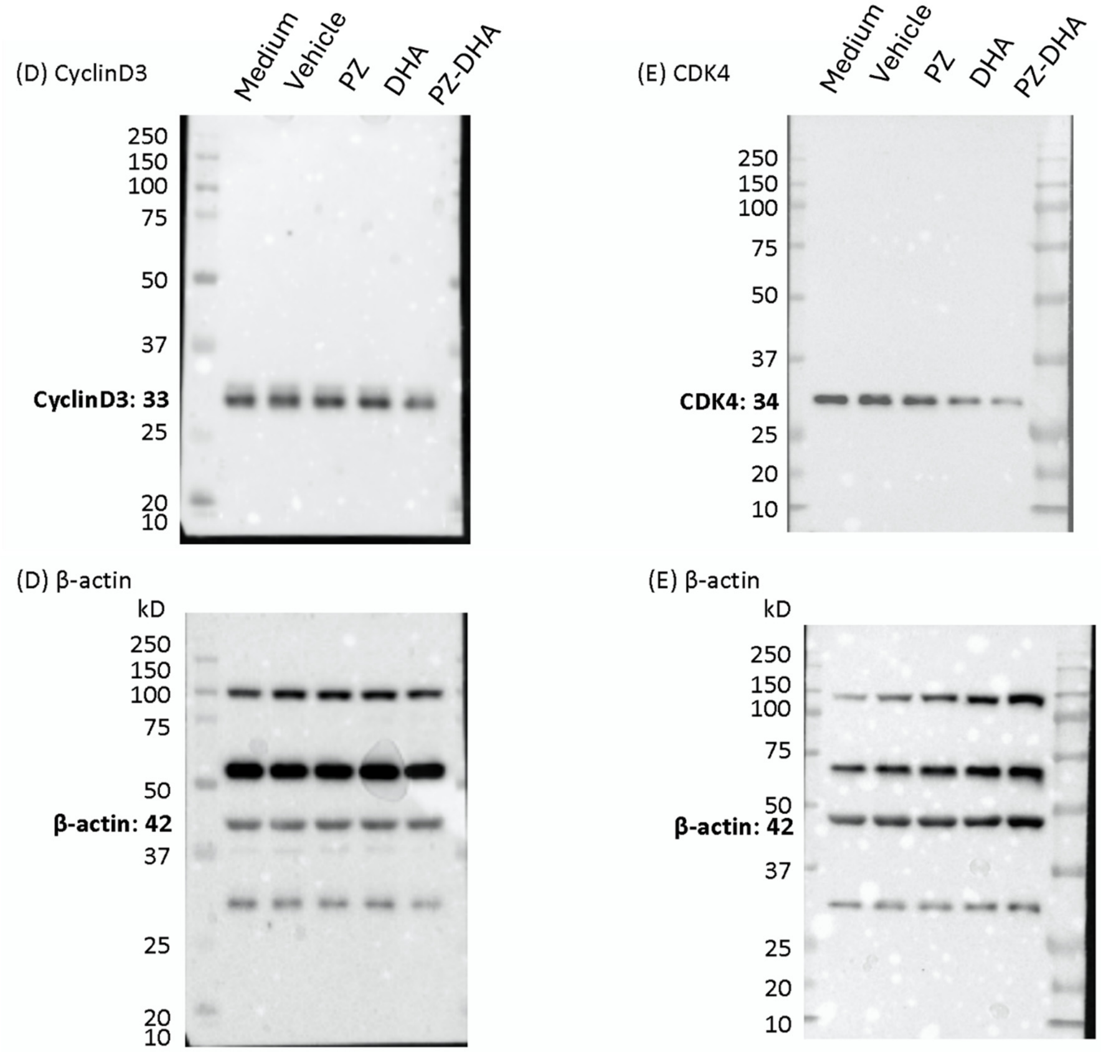

Figure S4. Original western blot images for Figure 3.

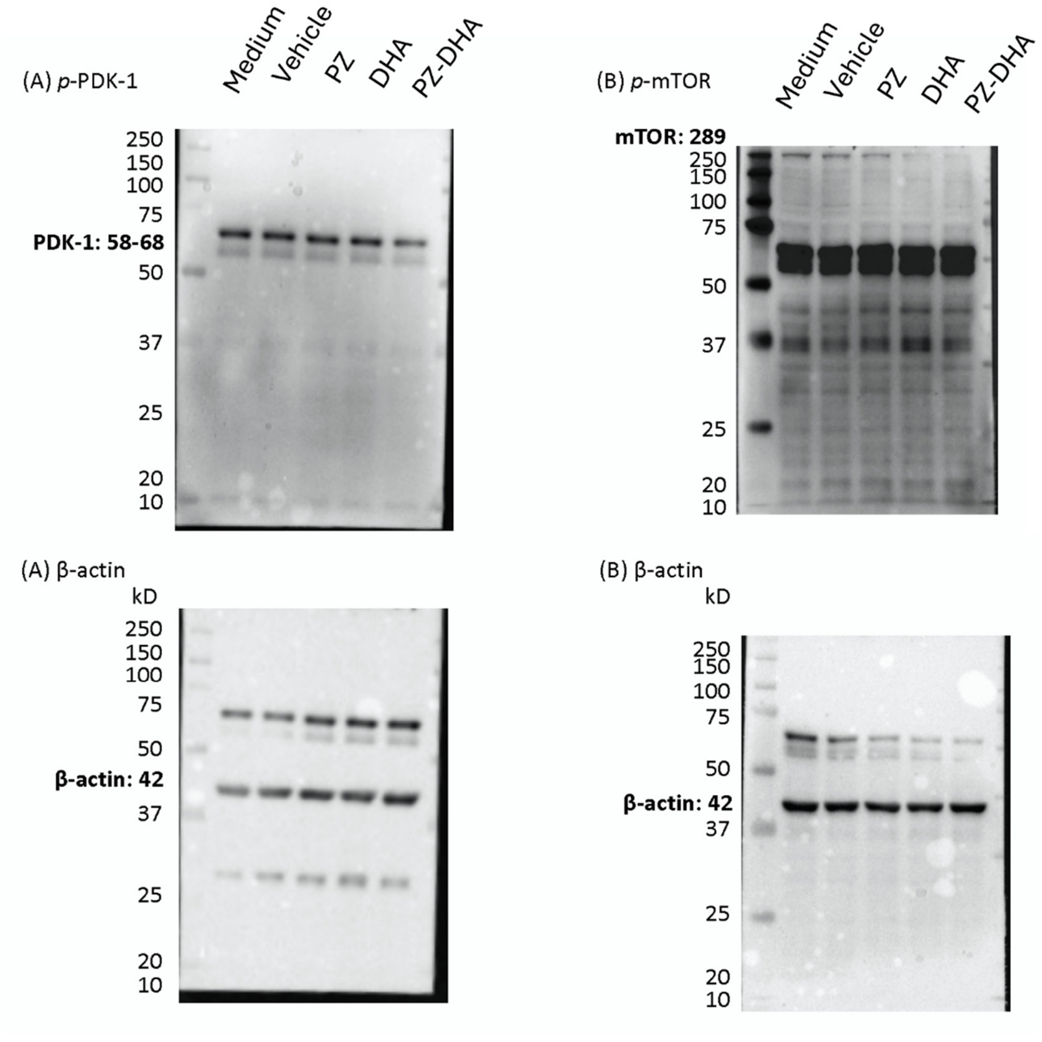

Figure S5. Original western blot images for Figure 5.

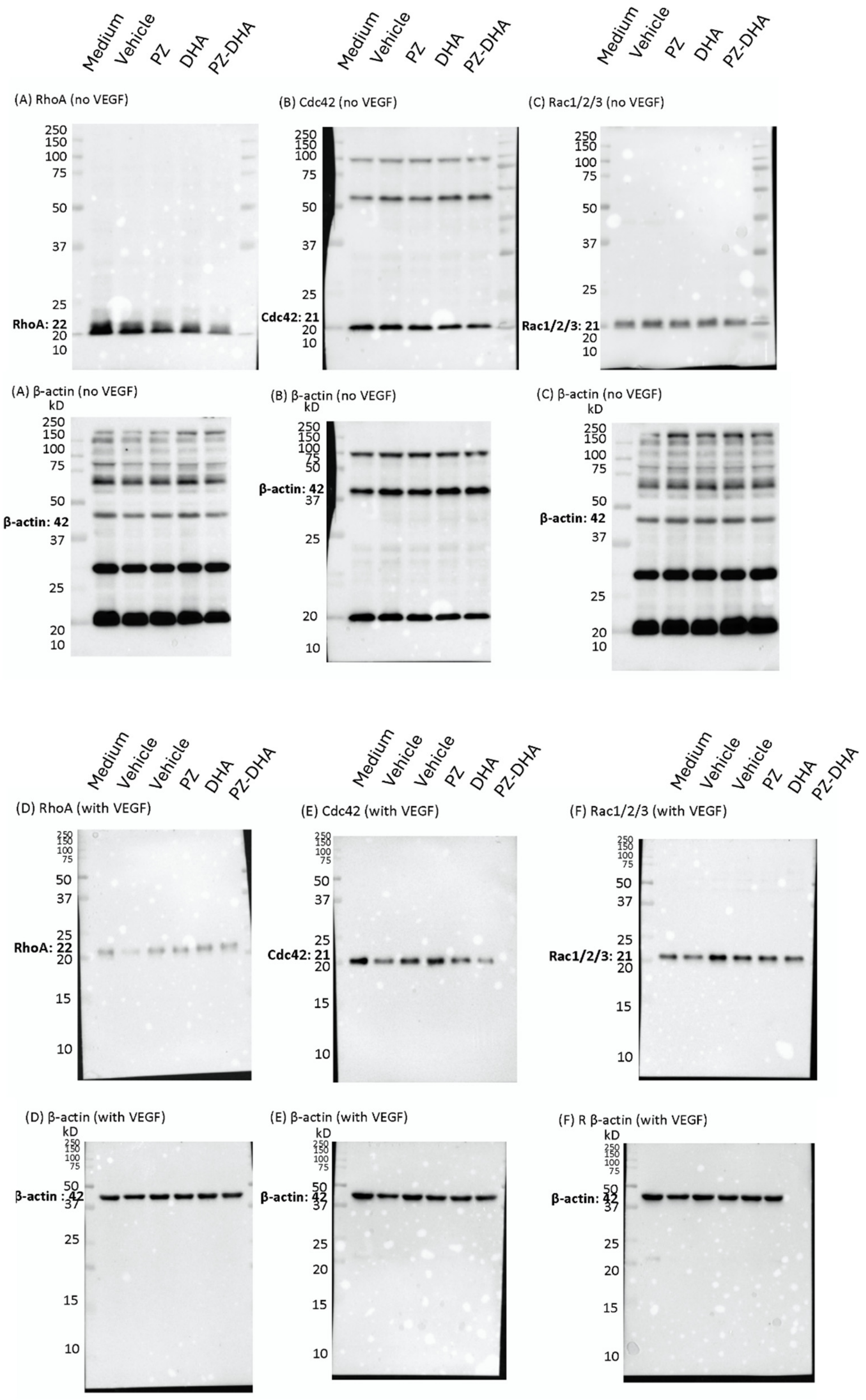

Figure S6. Original western blot images for Figure 6.
